# Toward targeted prevention: risk factors for prediabetes defined by impaired fasting glucose, impaired glucose tolerance and increased HbA1c in the population-based KORA study from Germany

**DOI:** 10.1007/s00592-020-01573-x

**Published:** 2020-08-03

**Authors:** Gregory G. Greiner, Karl M. F. Emmert-Fees, Jana Becker, Wolfgang Rathmann, Barbara Thorand, Annette Peters, Anne S. Quante, Lars Schwettmann, Michael Laxy

**Affiliations:** 1 Institute for Health Services Research and Health Economics, German Diabetes Center, Auf’m Hennekamp 65, 40225 Duesseldorf, Germany; 2 Medical Faculty, Centre for Health and Society, Institute for Health Services Research and Health Economics, Heinrich Heine University, Moorenstr. 5, 40225 Duesseldorf, Germany; 3 Institute of Health Economics and Health Care Management, Helmholtz Zentrum München GmbH, German Research Center for Environmental Health, Ingolstädter Landstraße 1, 85764 Neuherberg, Germany; 4 Institute and Outpatient Clinic for Occupational, Social and Environmental Medicine, University Hospital Munich, Ludwig-Maximilians-University Munich, Ziemssenstr. 1, 80336 Munich, Germany; 5 Institute for Biometry and Epidemiology, German Diabetes Center, Auf’m Hennekamp 65, 40225 Duesseldorf, Germany; 6 German Centre for Diabetes Research (DZD), Ingolstädter Landstraße 1, 85764 Munich-Neuherberg, Germany; 7 Institute of Epidemiology, Helmholtz Zentrum München GmbH, German Research Center for Environmental Health, Ingolstädter Landstraße 1, 85764 Neuherberg, Germany; 8 Institute of Genetic Epidemiology, Helmholtz Zentrum München, German Research Center for Environmental Health (GmbH), Ingolstädter Landstraße 1, 85764 Neuherberg, Germany; 9 Department of Gynecology and Obstetrics, Klinikum Rechts der Isar der Technischen Universität München, Ismaninger Str. 22, 81675 Munich, Germany; 10 Department of Economics, Martin Luther University Halle-Wittenberg, 06099 Halle (Saale), Germany; 11 Global Diabetes Research Center, Rollins School of Public Health, Emory University, 1518 Clifton Rd., NE, Atlanta, GA 30322 USA

**Keywords:** Prediabetes, Prevention, IGT, IFG, Increased HbA1c, Epidemiology

## Abstract

**Aims:**

To identify socioeconomic, behavioral and clinical factors that are associated with prediabetes according to different prediabetes definition criteria.

**Methods:**

Analyses use pooled data of the population-based Cooperative Health Research in the Region of Augsburg (KORA) studies (*n* = 5312 observations aged ≥ 38 years without diabetes). Prediabetes was defined through either impaired fasting glucose (IFG), impaired glucose tolerance (IGT) or elevated HbA1c according to thresholds of the American Diabetes Association. Explanatory variables were regressed on prediabetes using generalized estimating equations.

**Results:**

Mean age was 58.4 years; 50% had prediabetes (33% had IFG, 16% IGT, and 26% elevated HbA1c, 10% fulfilled all three criteria). Age, obesity, hypertension, low education, unemployment, statutory health insurance, urban residence and physical inactivity were associated with prediabetes. Male sex was a stronger risk factor for IFG (OR = 2.5; 95%–CI: 2.2–2.9) than for IGT or elevated HbA1c, and being unemployed was a stronger risk factor for IGT (OR = 3.2 95%–CI: 2.6–4.0) than for IFG or elevated HbA1c.

**Conclusions:**

The overlap of people with IFG, IGT and elevated HbA1c is small, and some factors are associated with only one criterion. Knowledge on sociodemographic and socioeconomic risk factors can be used to effectively target interventions to people at high risk for type 2 diabetes.

**Electronic supplementary material:**

The online version of this article (10.1007/s00592-020-01573-x) contains supplementary material, which is available to authorized users.

## Introduction

Diabetes is a burdensome and costly disease, which affects more than 420 million people worldwide and will affect 642 million in 2040 [1–6]. Around 90% of those people have type 2 diabetes mellitus (T2DM). In Germany, the prevalence of the disease continues to increase despite prevention efforts and disease management programs. More importantly, people with T2DM have two times higher direct and indirect medical costs than people without diabetes [7].

This situation is a great challenge for the financial sustainability of many healthcare systems across the globe and calls for effective and cost-effective T2DM prevention strategies. Decision-makers have multiple options among upstream to downstream interventions. Upstream interventions, for example, are regulatory, fiscal or environmental interventions that target risk factors of T2DM on the population level. In turn, downstream interventions often target high-risk individuals through clinical interventions. Whereas upstream interventions have a higher population impact and are more likely to be cost-effective than downstream interventions, the level of evidence for downstream interventions, such as individual lifestyle modification (LSM) interventions, is more robust [8]. The diabetes prevention program study in the USA, the Finish Diabetes Prevention Program, the Indian Diabetes Prevention Program, the Da Qing Diabetes Prevention study and many subsequent translational trials have shown that lifestyle interventions are effective in reducing weight and preventing onset in various populations at high risk for T2DM [9–12].

Economic evaluation studies show that LSM interventions are probably cost-effective in the long term. But they become less cost-effective if universal rather than targeted screening to identify people at high risk is applied or if interventions are offered to people with a lower diabetes risk [13–17]. Therefore, strategies to identify, inform and motivate individuals at high risk to get tested and to initiate lifestyle changes are core components to assure widespread adoption of interventions at reasonable costs. To steer and advise information campaigns and to tailor prevention initiatives to high-risk populations, more knowledge about their characteristics is needed.

The American Diabetes Association (ADA) defines individuals with a fasting plasma glucose (FPG) of 100–125 mg/dL [impaired fasting glucose (IFG)] or a 2-h postprandial glucose of 140–199 mg/dL [impaired glucose tolerance (IGT)] or an increased HbA1c [5.7%–6.4% (39–47 mmol/mol)] as having ‘prediabetes’ (i.e., intermediate hyperglycemia) and recommends preventive efforts in this population [18].

So far little is known about the characteristics of people with prediabetes in Europe. Furthermore, little is known about the potentially different characteristics and the overlap of the prediabetes groups as defined by IGT, IFG and increased HbA1c levels, as just a handful of studies gave a comparison of prevalence of prediabetes for all three criteria [19].

The aim of our study is therefore threefold. First, we investigate the overlap in populations that have prediabetes according to one of the three prediabetes criteria; second, we assess clinical, behavioral, sociodemographic and socioeconomic characteristics that are associated with prediabetes; and thirdly, we analyze whether those risk factors are the same for IGT, IFG and increased HbA1c levels.

## Research design and methods

### Population and study design

We used data from three studies of the population-based KORA (Cooperative Health Research in the Region of Augsburg) platform from Southern Germany. The study design of KORA, sampling methods and data collection have been described in detail elsewhere [20]. For our analyses, we pooled data from the population-based S4 study (1999–2001) which consisted of 4261 participants aged 24–74 years, and its two follow-up studies F4 (2006–2008, *n* = 3080) and FF4 (2013–2014, *n* = 2279). Study design, medical checkup, interviews and questionnaires of the three studies were very similar and, therefore, allowed pooling of these three study waves. As the prevalence of prediabetes in younger adults is low and to harmonize the samples from the different studies, we restricted our investigation to participants aged 38–79 leading to a total sample of *n* = 8005 observations (S4: *n* = 3110, F4: *n* = 2769, FF4: *n* = 2126).

To reflect a decision-maker perspective focusing on preventive efforts which aim at people with a high risk for diabetes, we excluded participants with known or newly diagnosed T2DM from the analysis sample (*n* = 925). We further excluded observations with missing values in one of the outcome variables FPG, 2-h postprandial glucose or HbA1c (including people < 55 years from the S4 study who did not receive an oGTT). This leads to a final analysis sample of *n* = 5312 observations across three time points (compare appendix Table S1). Hence, we obtained an analysis dataset with repeated observations including *n* = 1204 participants with one observation, *n* = 1595 persons with two observations and *n* = 306 people with three observations.

All three KORA studies were approved by the Ethics Committee of the Bavarian Medical Association. All study participants provided written informed consent.

### Measurements and definition of (pre-)diabetes

In all three studies, participants were asked to fast overnight and to avoid heavy physical activity on the day before the examination. People without known diabetes received a standard oGTT in the morning before the examination. HbA1c was measured based on capillary blood without exclusion criteria [21]. We used ADA criteria to define T2DM and prediabetes. Accordingly, participants with a previous T2DM diagnosis (known diabetes) or with FPG > 125 mg/dL, 2-h PG ≥ 200 mg/dL or HbA1c ≥ 6.5% (48 mmol/mol) were defined as having diabetes [22, 23]. Similarly, people with an FPG of 100–125 mg/dL (IFG), a 2-h postprandial glucose of 140–199 mg/dL (IGT) or an increased HbA1c 5.7–6.4% (39–47 mmol/mol) were defined as having prediabetes.

### Individual characteristics as explanatory factors

The choice of potential risk factors was guided by the literature [24]. We focused on sociodemographic and socioeconomic, clinical and behavioral parameters which are easily available in daily practice or routine data. This mimics the perspective and resources of health policy agencies.

We included sex, marital status (living with partner or not) and a 5-year categorization for age, in which the first and the last groups are covering more years for a better fitting group size. Individual socioeconomic status (SES) was characterized by educational level and equalized disposable income of the household. Education was classified based on educational years—low (less than 9 years), middle (9–12 years) and high (more than 12 years) levels of education. The equalized disposable income provided by the KORA studies is based on the midpoint of the self-reported net income group of the household and weighted relatively to the number and age of household members (weights of 1 for the head of the household, 0.8 for those aged 18 years and older, 0.9 for members aged 15–17 years, 0.65 for those aged 7–14 years and 0.5 for children in household aged ≤ 6 years). We created quintiles for our sample with quintile 1 (Q1) representing the highest equalized disposable income and quintile 5 (Q5) standing for the lowest equalized disposable income. In addition, three groups were categorized for employment status (full-time, part-time and marginal or irregular employed, not employed) and two groups for the type of health insurance (i.e., compulsory or private). In Germany, employees above a certain income level, but also self-employed persons or civil servants, can choose a full private health insurance instead of the compulsory one. We also took the place of residence (urban Augsburg city vs. rural district of Augsburg) into account.

With respect to clinical factors, obesity was defined as BMI ≥ 30 kg/m^2^, and a high waist circumference was specified as ≥ 102 cm for men and ≥ 88 cm for women [25]. The current status of hypertension was defined as having a systolic blood pressure ≥ 140 mmHg or/and a diastolic blood pressure ≥ 90 mmHg, or having diagnosed hypertension and/or taking anti-hypertensive medication given that the participants had known hypertension. Parental diabetes status (yes, no or unknown combined) was assessed and self-reported.

Regarding lifestyle factors, a sufficient level of physical activity was defined as performing physical exercise at least 60 min/week regularly. Low-risk gender-specific alcohol intake was assumed following the criteria of the Federal Centre for Health Education by setting cut points at ≤ 24 g/day for men and ≤ 12 g/day for women [26]. Finally, self-reported smoking status was categorized as never smoker, ex-smoker and current smoker.

### Statistical analyses

The pooled sample was treated as a cross-sectional dataset in all analyses, and multivariable analyses accounted for the nested structure. We chose this pragmatic approach since in this work we are not interested in the longitudinal effects of the risk factors but to increase the power of our analyses. In a first analysis step, we described the prevalence as well as the overlap of people with prediabetes according to the three prediabetes criteria (IFG, IGT and increased HbA1c levels) using a proportional Venn diagram. In a second step, we regressed the explanatory factors on prediabetes defined by the three criteria separately and combined. For each of the four outcomes, we fitted both simple models to investigate each explanatory factor separately and multivariable models to test all explanatory variables simultaneously. We used generalized estimating equation (GEE) models with a binary distribution using a logit link and a compound symmetry covariance structure to account for the nested structure of the pooled analysis sample. For all analyses, missing data of explanatory variables were imputed using Markov Chain Monte Carlo procedures (*n* = 5 imputations, an overview of missing patterns is given in Table S2 in Appendix). All data analyses were performed using SAS V.9.4 (SAS Institute). The results for the analyses of the imputed samples were combined using the SAS procedure MIANALYZE.

## Results

### Sample characteristics and prevalence of diabetes and prediabetes

A summary on the characteristics of participants with and without prediabetes is presented in Table 1. The mean age was 58.4. About 33% of all participants had IFG, 16% had IGT and for 26% an increased HbA1c level was observed. Following the suggestion of ADA to consider any of the three criteria to define prediabetes, the prevalence was 50%.

**Table 1:** Characteristics of the population without diabetes

Variables	All (*n* = 5312, 100%)		HbA1c > 5.7% (*n* = 1357, 25.5%)		IFG (*n* = 1775, 33.4%)		IGT (*n* = 842, 15.8%)		Prediabetes^a^ (*n* = 2658, 50%)		Normal^b^ (*n* = 2654, 50%)	
	*n*/mean	%/SD	*n*/mean	%/SD	*n*/mean	%/SD	*n*/mean	%/SD	*n*/mean	%/SD	*n*/mean	%/SD
KORA study												
S4	1200	22.59	497	36.62	520	29.30	238	28.27	822	30.93	378	14.24
F4	2358	44.39	559	41.19	633	35.66	358	42.52	1023	38.49	1335	50.30
FF4	1754	33.02	301	22.18	622	35.04	246	29.22	813	30.59	941	35.46
Sex [male]	2543	47.87	640	47.16	1115	62.82	437	51.90	1453	54.67	1090	41.07
Marital status [living alone]	1310	24.66	369	27.19	424	23.89	207	24.58	680	25.58	630	23.74
Age	58.45	10.67	63.29	8.64	61.83	9.36	64.36	9.13	62.17	9.39	54.72	10.57
38–44 years	705	13.27	40	2.95	97	5.46	33	3.92	140	5.27	565	21.29
45–49 years	592	11.14	66	4.86	126	7.10	34	4.04	169	6.36	423	15.94
50–54 years	567	10.67	88	6.48	139	7.83	52	6.18	211	7.94	356	13.41
55–59 years	867	16.32	231	17.02	293	16.51	101	12.00	437	16.44	430	16.20
60–64 years	878	16.53	291	21.44	378	21.30	160	19.00	546	20.54	332	12.51
65–69 years	753	14.18	272	20.04	326	18.37	181	21.50	494	18.59	259	9.76
70–79 years	950	17.88	369	27.19	416	23.44	281	33.37	661	24.87	289	10.89
Educational status												
Less than 9 years	520	9.79	200	14.74	178	10.03	127	15.08	326	12.26	194	7.31
9–12 years	3123	58.79	858	63.23	1110	62.54	506	60.10	1651	62.11	1472	55.46
More than 12 years	1659	31.23	297	21.89	485	27.32	207	24.58	675	25.40	984	37.08
Equivalent household income												
Quintile 1 (high)	1004	18.90	234	17.24	355	20.00	149	17.70	489	18.40	515	19.40
Quintile 2	971	18.28	242	17.83	348	19.61	134	15.91	486	18.28	485	18.27
Quintile 3	1173	22.08	292	21.52	383	21.58	200	23.75	592	22.27	581	21.89
Quintile 4	981	18.47	263	19.38	323	18.20	163	19.36	492	18.51	489	18.43
Quintile 5 (low)	928	17.47	262	19.31	288	16.23	154	18.29	476	17.91	452	17.03
Employment status												
Full-time	1790	33.70	306	22.55	553	31.15	155	18.41	734	27.61	1056	39.79
Regular part-time, marginal or irregular employed	1866	35.13	540	39.79	581	32.73	291	34.56	938	35.29	928	34.97
Not employed	1385	26.07	400	29.48	543	30.59	317	37.65	800	30.10	585	22.04
Health insurance [statutory]	4314	81.21	1160	85.48	1437	80.96	730	86.70	2211	83.18	2103	79.24
Residence [urban]	2233	42.04	625	46.06	791	44.56	350	41.57	1206	45.37	1027	38.70
BMI [≥ 30]	1308	24.62	455	33.53	629	35.44	352	41.81	882	33.18	426	16.05
Waist circumference [high (sex-specific)]	2358	44.39	771	56.82	1009	56.85	562	66.75	1469	55.27	889	33.50
Hypertension [yes]	2000	37.65	711	52.39	928	52.28	518	61.52	1346	50.64	654	24.64
Parental diabetes [yes]	1430	26.92	377	27.78	537	30.25	247	29.33	777	29.23	653	24.60
Physical activity [less than 1 h/week]	2326	43.79	678	49.96	847	47.72	427	50.71	1274	47.93	1052	39.64
Alcohol consumption [high consumption (sex-specific)]	1671	31.46	385	28.37	633	35.66	279	33.14	886	33.33	785	29.58
Smoking status												
Current smoker	890	16.75	223	16.43	241	13.58	90	10.69	385	14.48	505	19.03
Ex-smoker	2131	40.12	521	38.39	804	45.30	355	42.16	1122	42.21	1009	38.02
Never smoker	2287	43.05	613	45.17	729	41.07	397	47.15	1150	43.27	1137	42.84

*IFG* impaired fasting glucose (100–125 mg/dL); *IGT* impaired glucose tolerance (140–199 mg/dL); *SD* standard deviation

^a^HbA1c > 5.7% or IFG or IGT

^b^No HbA1c > 5.7%, IFG and IGT

### Overlap in populations with prediabetes defined by different criteria

**Fig. 1:**
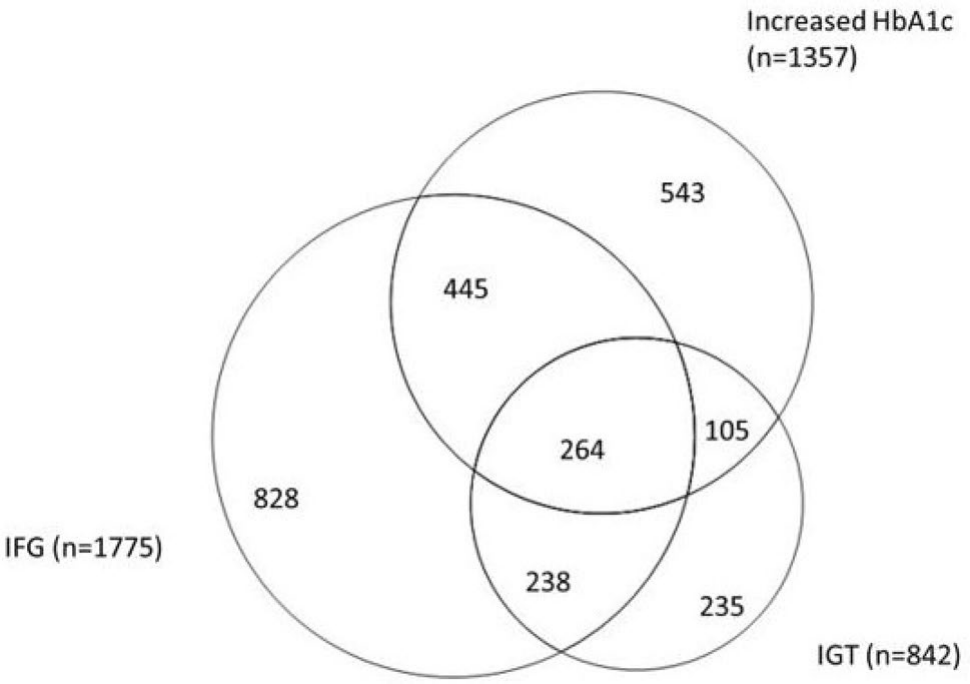
Proportional Venn diagram showing the overlap of the prediabetes criteria

The proportional Venn diagram (Fig. 1) presents the joint distribution of observations with IFG, IGT and increased HbA1c levels. Only 264 (9.6%) of 2658 people with prediabetes fulfilled all three criteria, whereas 788 (29.6%) satisfied two of them. The largest overlap was between IFG and IGT and the smallest overlap between IGT and increased HbA1c.

Sex-stratified analyses showed that men were more likely than women to be categorized with prediabetes via the IFG criterion, whereas women were mostly classified as in prediabetes state with the HbA1c criterion (Figure S1 in Appendix).

### Risk factors for prediabetes—univariate analyses

Table 2 shows the univariate results for the analyzed explanatory factors as odds ratios. We found that being male (OR = 1.76; 95%–CI: 1.55–1.99), higher age (OR = 9.90; 95%–CI: 7.84–12.50 for the oldest group vs. the youngest age-group), low levels of education (OR = 2.61; 95%–CI: 2.08–3.29), not employed (OR = 1.86; 95%–CI: 1.62–2.14), statutory health insurance (no private though) (OR = 1.32; 95%–CI: 1.13–1.55), living in urban areas (OR = 1.28; 95%–CI: 1.13–1.45), obesity (OR = 2.54; 95%–CI: 2.21–2.93), high waist circumference (OR = 2.32; 95%–CI: 2.06–2.61), hypertension (OR = 2.89; 95%–CI: 2.55–3.27), parental diabetes (OR = 1.31; 95%–CI: 1.14–1.51), physical inactivity (OR = 1.35; 95%–CI: 1.21–1.51) and high alcohol consumption (OR = 1.18; 95%–CI: 1.05–1.33) significantly increased the likelihood for having prediabetes according to combined criteria. In contrast, living alone, the income level and smoking behavior were not associated with an increased likelihood for having prediabetes. Contrary associations seen with smoking behaviour are mainly due differences in age.

**Table 2:** Risk factors for prediabetes—univariate models

	HbA1c > 5.7%		IFG		IGT		Any criterion	
	OR	95%–CI	OR	95%–CI	OR	95%–CI	OR	95%–CI
KORA study (reference: S4)								
F4	0.51	0.44–0.58	0.61	0.54–0.69	0.87	0.73–1.03	0.45	0.40–0.51
FF4	0.39	0.33–0.45	1.06	0.93–1.21	0.88	0.73–1.06	0.59	0.51–0.68
Sex [male]	0.99	0.86–1.14	2.49	2.18–2.86	1.22	1.04–1.44	1.76	1.55–1.99
Marital status [living alone]	1.17	1.01–1.36	0.98	0.85–1.14	1.02	0.85–1.22	1.12	0.98–1.29
Age (reference: 38–44 years)								
45–49 years	2.10	1.45–3.05	1.89	1.47–2.45	1.31	0.83–2.05	1.76	1.40–2.21
50–54 years	3.06	2.11–4.44	2.35	1.79–3.08	2.15	1.40–3.32	2.61	2.06–3.31
55–59 years	6.19	4.40–8.72	3.77	2.93–4.85	2.66	1.78–3.97	4.59	3.67–5.73
60–64 years	7.79	5.55–10.95	5.11	3.97–6.57	4.44	3.02–6.53	6.78	5.41–8.50
65–69 years	9.03	6.41–12.73	5.63	4.36–7.27	6.54	4.46–9.58	8.16	6.45–10.31
70–79 years	9.86	7.03–13.83	6.27	4.87–8.07	9.18	6.32–13.35	9.90	7.84–12.50
Educational status (reference: more than 12 years)								
Less than 9 years	2.93	2.30–3.73	1.35	1.06–1.71	2.32	1.77–3.05	2.61	2.08–3.29
9–12 years	1.79	1.51–2.12	1.39	1.19–1.62	1.40	1.15–1.70	1.69	1.47–1.94
Equivalent household income (reference: highest quintile)								
Quintile 2	1.11	0.91–1.35	1.10	0.92–1.31	0.93	0.73–1.19	1.10	0.93–1.31
Quintile 3	1.03	0.85–1.26	1.00	0.84–1.19	1.23	0.98–1.55	1.09	0.92–1.29
Quintile 4	1.13	0.92–1.39	0.94	0.78–1.13	1.14	0.90–1.45	1.02	0.85–1.22
Quintile 5 (low)	1.23	0.99–1.53	0.90	0.74–1.09	1.16	0.91–1.49	1.11	0.92–1.33
Employment status (reference: full time)								
Regular part-time, marginal or irregular employed	1.89	1.61–2.22	1.13	0.98–1.30	2.02	1.65–2.49	1.53	1.34–1.75
Not employed	1.82	1.53–2.16	1.47	1.27–1.70	3.20	2.58–3.97	1.86	1.62–2.14
Health insurance [statutory]	1.53	1.26–1.85	1.01	0.85–1.20	1.60	1.27–2.03	1.32	1.13–1.55
Residence [urban]	1.22	1.06–1.40	1.12	0.98–1.28	0.98	0.83–1.15	1.28	1.13–1.45
BMI [≥ 30]	1.83	1.58–2.11	2.30	2.00–2.64	2.64	2.24–3.12	2.54	2.21–2.93
Waist circumference [high (sex-specific)]	1.88	1.64–2.14	2.09	1.86–2.36	2.92	2.48–3.44	2.32	2.06–2.61
Hypertension [yes]	2.19	1.91–2.50	2.36	2.08–2.68	3.08	2.63–3.61	2.89	2.55–3.27
Parental diabetes [yes]	1.07	0.92–1.26	1.32	1.14–1.53	1.18	0.99–1.41	1.31	1.14–1.51
Physical activity [less than 1 h/week]	1.37	1.21–1.56	1.22	1.08–1.37	1.29	1.11–1.51	1.35	1.21–1.51
Alcohol consumption [high consumption (sex-specific)]	0.85	0.74–0.97	1.24	1.10–1.40	1.13	0.97–1.32	1.18	1.05–1.33
Smoking status (reference: never smoker)								
Current smoker	0.92	0.76–1.12	0.78	0.64–0.94	0.54	0.41–0.70	0.75	0.63–0.89
Ex-smoker	0.90	0.77–1.04	1.31	1.14–1.52	0.99	0.83–1.18	1.14	0.99–1.31

Results are based on a generalized estimating equation (GEE) with prediabetes (HbA1c > 5.7%, IGT, IFG) vs. no prediabetes as binary outcome; analysis sample *n* = 5312

*IFG* impaired fasting glucose (100–125 mg/dL); *IGT* impaired glucose tolerance (140–199 mg/dL); *OR* odds ratio; *CI* confidence interval; [], tested category; (), reference category

Generally, the associations between explanatory variables and prediabetes according to the three different criteria were similar. However, a few factors stood out: male sex increased the likelihood for having IFG substantially (OR = 2.49; 95%–CI: 2.18–2.86), but not for increased HbA1c levels (OR = 0.99; 95%–CI: 0.86–1.14) and only moderately for IGT (OR = 1.22; 95%–CI: 1.04–1.44). In addition, unemployment was more strongly associated with IGT (OR = 3.20; 95%–CI: 2.58–3.97) than it was with IFG (OR = 1.47; 95%–CI: 1.27–1.70) or increased HbA1c (OR = 1.82; 95%–CI: 1.53–2.16).

### Risk factors for prediabetes—multivariate results

The results of the multivariate analyses are shown in Table 3. Being male, higher age, living in urban areas, obesity, waist circumference, hypertension and parental diabetes also increased the likelihood for prediabetes according to the combined criteria in the adjusted model. However, the effect estimates for education, employment, health insurance, residency and physical inactivity were substantially smaller than in the univariate model and in most cases no longer significant. As in the univariate models, male sex was stronger associated with IFG than with IGT and increased HbA1c levels, and unemployment had a much higher association with IGT than with IFG or increased HbA1c levels.

**Table 3:** Risk factors for prediabetes—multivariate models

	HbA1c > 5.7%		IFG		IGT		Any criterion	
	OR	95%–CI	OR	95%–CI	OR	95%–CI	OR	95%–CI
KORA study (reference: S4)								
F4	0.68	0.56–0.84	0.68	0.57–0.82	1.06	0.82–1.36	0.56	0.46–0.68
FF4	0.43	0.35–0.53	1.10	0.90–1.34	0.97	0.74–1.27	0.64	0.52–0.79
Sex [male]	0.96	0.81–1.14	2.70	2.29–3.18	1.38	1.13–1.68	1.94	1.66–2.27
Marital status [living alone]	0.98	0.83–1.16	0.96	0.82–1.14	0.85	0.70–1.04	1.01	0.86–1.19
Age (reference: 38–44 years)								
45–49 years	2.23	1.53–3.24	1.56	1.20–2.04	1.19	0.76–1.85	1.55	1.22–1.97
50–54 years	3.06	2.08–4.49	1.85	1.40–2.46	1.81	1.17–2.79	2.19	1.70–2.82
55–59 years	4.72	3.29–6.78	2.56	1.93–3.38	1.92	1.26–2.92	2.90	2.26–3.72
60–64 years	6.04	4.15–8.77	3.17	2.37–4.25	2.71	1.76–4.17	3.99	3.06–5.19
65–69 years	6.83	4.61–10.13	3.35	2.45–4.57	3.39	2.16–5.32	4.56	3.41–6.08
70–79 years	8.32	5.61–12.33	3.59	2.62–4.92	4.59	2.94–7.17	5.70	4.25–7.65
Educational status (reference: more than 12 years)								
Less than 9 years	1.11	0.83–1.49	1.07	0.79–1.44	1.00	0.70–1.41	1.22	0.91–1.63
9–12 years	1.17	0.97–1.43	1.23	1.02–1.47	0.90	0.72–1.13	1.23	1.04–1.45
Equivalent household income (reference: highest quintile)								
Quintile 2	1.09	0.87–1.36	1.00	0.82–1.22	0.84	0.65–1.10	1.03	0.85–1.26
Quintile 3	0.93	0.74–1.16	0.94	0.77–1.15	0.96	0.75–1.25	1.00	0.82–1.22
Quintile 4	0.91	0.72–1.16	0.92	0.75–1.14	0.82	0.62–1.08	0.91	0.73–1.13
Quintile 5 (low)	1.09	0.85–1.40	0.84	0.67–1.04	0.82	0.62–1.10	1.00	0.80–1.25
Employment status (reference: full time)								
Regular part-time, marginal or irregular employed	0.91	0.73–1.13	0.93	0.76–1.12	1.30	0.99–1.70	0.95	0.79–1.15
Not employed	0.96	0.76–1.21	1.07	0.85–1.33	1.53	1.12–2.10	1.10	0.89–1.35
Health insurance [statutory]	1.12	0.89–1.40	0.94	0.77–1.16	1.38	1.04–1.82	1.08	0.89–1.31
Residence [urban]	1.14	0.98–1.32	1.09	0.94–1.26	0.94	0.78–1.12	1.25	1.08–1.44
BMI [≥ 30]	1.26	1.04–1.51	1.60	1.34–1.91	1.62	1.30–2.00	1.66	1.37–2.01
Waist circumference [high (sex-specific)]	1.38	1.17–1.64	1.50	1.28–1.76	1.78	1.44–2.20	1.53	1.30–1.80
Hypertension [yes]	1.37	1.18–1.59	1.47	1.27–1.69	1.71	1.43–2.04	1.60	1.39–1.85
Parental diabetes [yes]	1.16	0.98–1.38	1.37	1.16–1.61	1.27	1.04–1.54	1.45	1.24–1.69
Physical activity [less than 1 h/week]	1.10	0.95–1.26	1.07	0.93–1.22	1.07	0.90–1.27	1.10	0.97–1.26
Alcohol consumption [high consumption (sex-specific)]	0.80	0.69–0.93	1.14	1.00–1.31	1.11	0.94–1.32	1.11	0.97–1.27
Smoking status (reference: never smoker)								
Current smoker	1.40	1.13–1.74	0.83	0.67–1.03	0.83	0.62–1.11	0.96	0.79–1.17
Ex-smoker	0.95	0.80–1.12	1.00	0.85–1.17	0.91	0.75–1.10	1.00	0.85–1.16

Results are based on a generalized estimating equation (GEE) with prediabetes (HbA1c > 5.7%, IGT, IFG) vs. no prediabetes as binary outcome; analysis sample *n* = 5312

*IFG* impaired fasting glucose (100–125 mg/dL); *IGT* impaired glucose tolerance (140–199 mg/dL); *OR* odds ratio; *CI* confidence interval; [], tested category; (), reference category

## Discussion

### Summary

In order to be cost-effective, downstream information campaigns and interventions aiming to prevent T2DM must effectively target people at high risk. Hence, we analyzed which sociodemographic, socioeconomic, behavioral and clinical factors are associated with prediabetes. Furthermore, we analyzed the overlap of the three prediabetes criteria and whether the risk factors for IFG, IGT and increased HbA1c levels differed. We observed that the overlap of people defined through all three prediabetes criteria is quite small and that age, obesity, hypertension, low levels of education, unemployment, statutory health insurance, living in urban areas and physical inactivity are risk factors for prediabetes. We also found that some risk factors for the three prediabetes stages differed. For example, men are more likely to have IFG than women, whereas women are more likely to have IGT or increased HbA1c levels. Similarly, unemployment is strongly associated with IGT, but only weakly with IFG or increased HbA1c levels.

### Comparison with previous studies

To our knowledge, no previous study comprehensively described the overlap of all three criteria (IGT, IFG and increased HbA1c levels) in a large European population-based sample. A recent review from Barry et al. identified only five studies that compared IGT, IFG and increased HbA1c levels in one sample but only two of those studies (one from China, one from the USA) were based on population-based samples. The pooled data of the five studies showed that the prevalence of prediabetes with ADA criteria was 54% and 8.7% of those with prediabetes fulfilled all three criteria [19]. Similarly, Saukkonen et al. reported in a small Finish sample that the overlap for HbA1c > 5.7%, IFG and IGT in people with prediabetes was quite small [27]. In that study, 34% of participants were classified as having prediabetes and only 3% of those with prediabetes fulfilled all three prediabetes criteria. With 10%, the overlap of people with prediabetes who had increased HbA1c levels, IFG and IGT in our study was comparably small. Furthermore, comparable to the study of Barry et al., the majority of people with prediabetes in our sample had IFG (67%) and increased HbA1c (51%), whereas the prevalence of IGT (32%) was much lower. That the joint distribution of IGT, IFG and increased HbA1c differs significantly between men and women with a much higher proportion of women with increased HbA1c values is a new finding that has not been reported in this way before. The reasons for this finding are unknown, but the data show that the choice of the definition for prediabetes is likely to have a large impact on the share of women and men that are having prediabetes and might be eligible for certain types of lifestyle interventions to prevent diabetes.

There are also few studies that analyzed the full range of clinical, behavioral, sociodemographic and socioeconomic factors that are associated with prediabetes. Similar to our study, a cross-sectional study based on a Spanish sample showed that the modifiable risk factors alcohol consumption, hypertension and weight and lipid status are associated with prediabetes defined through IFG or HbA1c > 5.7% [28]. Other studies found that low income and education levels or living in deprived areas are associated with the existence of T2DM, but only few investigations are available that analyze factors associated with prediabetes [29–32].

We did not find studies that explicitly compared the characteristics of people with IFG, IGT and increased HbA1c values. Measurements of fasting glucose, 2-h postprandial glucose and HbA1c have different advantages in terms of practicability and costs. Furthermore, both the transition probability from prediabetes to diabetes and the relative risk reduction that can be managed through lifestyle interventions differ between people with IGT, IFG and increased HbA1c [19, 33]. Therefore, knowledge on the risk factors of corresponding high-risk groups is highly valuable to choose the best suitable diagnostic criteria and to identify the right target groups for specific diabetes prevention approaches.

### Implications for health policy

Several countries have initiated large-scale programs to promote and deliver LSM interventions, i.e., diabetes prevention programs, to individuals at high risk. Since the initiation of the National Diabetes Prevention Program in the USA, a public–private partnership to implement low-cost intervention (LCI) diabetes prevention programs in community setting, more than 240,000 people at high risk have been enrolled into one of the programs [34]. However, given that more than 80 million Americans have prediabetes, only a small fraction of at-risk individuals has received lifestyle interventions [35]. The gap in the cascade of diabetes prevention has also been highlighted in a recent analysis showing that only around a third of people with prediabetes have been told by their doctors that they are at high risk [36]. Therefore, reaching people at high risk to attend regular screening procedures and to engage in healthy lifestyle is of great importance for a successful implementation of large-scale diabetes prevention programs or efforts for high-risk individuals—particularly as targeted screening and identification of high-risk individuals are more cost-effective than universal screening [37].

One instrument to reach specific populations is media campaigns [38, 39]. Although media campaigns can potentially approach large segments of the population, even these methods can be optimized by correctly addressing the population subgroups at high risk for T2DM. In contrast, to target physician–patient communication guided by clinical variables, health media campaigns rely on data available to public health advocates such as information on sociodemographic and socioeconomic background of groups. The Federal Centre for Health Education (BZgA) in Germany recently initiated an information and communication strategy to prevent and treat T2DM [40]. The results of our study are very valuable for such national efforts. For example, our findings indicate that age is one of the strongest risk factors and prevention efforts in elderly settings will reach many high-risk individuals. Furthermore, our study shows that information campaigns aiming to raise awareness for prediabetes might be best targeted to statutorily insured people, those living in urban areas or visiting job centers, working in the blue collar industry where the proportion of university graduates is low or working in other industry sectors where physical activity levels are typically low.

### Strengths and limitations

This is one of the first studies testing the associations of a broad set of sociodemographic, socioeconomic, clinical and behavioral factors with prediabetes in a large European sample. A strength of this study is its population-based design with standardized measures of FPG, 2 h-PGG and HbA1c. Furthermore, using a pragmatic health policy perspective and the use of easy-to-measure characteristics as potential predictors allow physicians and health agencies to target screening, prevention and information campaigns.

As a limitation, it needs to be acknowledged that the data we used were sampled from a relatively affluent region in Southern Germany, where people are more likely to be healthier compared to the average German population. Furthermore, due to the design of our pooled analysis of cohort data and the likelihood of selective attrition toward more healthy participants in the follow-up studies, it is likely that the prevalence of prediabetes is underestimated in our analysis. However, it is unlikely that this biased the analyzed associations. Finally, although the data come from a population-based study, the analysis sample is not fully age representative as no OGTT was performed in people < 55 years in the baseline examination.

### Conclusions

Knowledge on risk factors for prediabetes is important to effectively target high-risk individuals with downstream prevention approaches. This study shows that besides clinical and behavioral factors, also easily available sociodemographic and socioeconomic data can be used to inform this process. Importantly, it should be acknowledged that the overlap in people with IGT, IFG and increased HbA1c levels is small and that these groups differ in certain characteristics.

## Electronic supplementary material

Below is the link to the electronic supplementary material.

Supplementary file1 (DOCX 253 kb)

## Data Availability

KORA data used in this study can be applied for via the digital application tool KORA.PASST as part of a project agreement under https://epi.helmholtz-muenchen.de/.
