# Supplementary material for: Toward targeted prevention: risk factors for prediabetes defined by impaired fasting glucose, impaired glucose tolerance and increased HbA1c in the population-based KORA study from Germany
Source: Acta Diabetol. 2020 Aug 3;57(12):1481–91. doi: 10.1007/s00592-020-01573-x (PMC7591423; doi:10.1007/s00592-020-01573-x)

| **Table S1: Overview on study samples** | | | | | | | | | | | | | | | | | | |
| --- | --- | --- | --- | --- | --- | --- | --- | --- | --- | --- | --- | --- | --- | --- | --- | --- | --- | --- |
|  | Total | | With diabetes | | No Diabetes | | No diabetes  (HbA1c>5.7%, IFG & IGT) | | HbA1c>5.7% | | IFG | | IGT | | HbA1c>5.7% or IFG or IGT | | No HbA1c>5.7% and IFG and IGT | |
| study | n | % | n | % | n | % | n | % | n | % | n | % | n | % | n | % | n | % |
| S4 (3110) | 3110 | 100.0 | 308 | 9.9 | 2802 | 90.1 | 1200 | 100.0 | 497 | 41.4 | 520 | 43.3 | 238 | 19.8 | 822 | 68.5 | 378 | 31.5 |
| S4<55 years (1467)* | 1467 | 100.0 | 37 | 2.5 | 1430 | 97.5 | 0 |  |  |  |  |  |  |  |  |  |  |  |
| S4>=55 years (1643) | 1643 | 100.0 | 271 | 16.5 | 1372 | 83.5 | 1200 | 100.0 | 497 | 41.4 | 520 | 43.3 | 238 | 19.8 | 822 | 68.5 | 378 | 31.5 |
|  |  |  |  |  |  |  |  |  |  |  |  |  |  |  |  |  |  |  |
| F4 (2769) | 2769 | 100.0 | 336 | 12.1 | 2433 | 87.9 | 2358 | 100.0 | 559 | 23.7 | 633 | 26.8 | 358 | 15.2 | 1023 | 43.4 | 1335 | 56.6 |
| F4 <55 years (1174) | 1174 | 100.0 | 35 | 3.0 | 1139 | 97.0 | 1095 | 46.4 | 125 | 11.4 | 176 | 16.1 | 73 | 6.7 | 283 | 25.8 | 812 | 74.2 |
| F4>=55 years (1595) | 1595 | 100.0 | 301 | 18.9 | 1294 | 81.1 | 1263 | 53.6 | 434 | 34.4 | 457 | 36.2 | 285 | 22.6 | 740 | 58.6 | 523 | 41.4 |
|  |  |  |  |  |  |  |  |  |  |  |  |  |  |  |  |  |  |  |
| FF4 (2126) | 2126 | 100.0 | 281 | 13.2 | 1845 | 86.8 | 1754 | 100.0 | 301 | 17.2 | 622 | 35.5 | 246 | 14.0 | 813 | 46.4 | 941 | 53.6 |
| FF4 <55 years (843) | 843 | 100.0 | 30 | 3.6 | 813 | 96.4 | 769 | 43.8 | 69 | 9.0 | 186 | 24.2 | 46 | 6.0 | 237 | 30.8 | 532 | 69.2 |
| FF4>=55 years (1283) | 1283 | 100.0 | 251 | 19.6 | 1032 | 80.4 | 985 | 56.2 | 232 | 23.6 | 436 | 44.3 | 200 | 20.3 | 576 | 58.5 | 409 | 41.5 |
|  |  |  |  |  |  |  |  |  |  |  |  |  |  |  |  |  |  |  |
| Total (8005) | 8005 | 100.0 | 925 | 11.6 | 7080 | 88.4 | **5312** | **100.0** | **1357** | **25.5** | **1775** | **33.4** | **842** | **15.9** | **2658** | **50.0** | **2654** | **50.0** |
| Total <55 years (3484) | 3484 | 100.0 | 102 | 2.9 | 3382 | 97.1 | 1864 | 35.1 | 194 | 10.4 | 362 | 19.4 | 119 | 6.4 | 520 | 27.9 | 1344 | 72.1 |
| Total >=55 years (4521) | 4521 | 100.0 | 823 | 18.2 | 3698 | 81.8 | 3448 | 64.9 | 1163 | 33.7 | 1413 | 41.0 | 723 | 21.0 | 2138 | 62.0 | 1310 | 38.0 |

* no OGGT was performed in this age group
IFG: impaired fasting glucose (100-125mg/dL); IGT: impaired glucose tolerance (140-199mg/dL)

| **Table S2: Effects of the multiple imputation on the statistical sample** | | | | | | | |
| --- | --- | --- | --- | --- | --- | --- | --- |
|  | **Before imputation** | | |  | **After imputation** | | |
| **Categorical variable** | N missing | mean of category | standard deviation |  | N missing | mean of category | standard deviation |
| Marital status | 1 | 0.75 | 0.43 |  | 0 | 0.75 | 0.43 |
| Educational status | 10 | 1.21 | 0.60 |  | 0 | 1.21 | 0.60 |
| Equivalent household income | 255 | 2.97 | 1.38 |  | 0 | 2.98 | 1.37 |
| Employment status | 271 | 1.08 | 0.79 |  | 0 | 1.05 | 0.79 |
| Health insurance | 36 | 0.82 | 0.39 |  | 0 | 0.82 | 0.39 |
| Residence | 18 | 0.42 | 0.49 |  | 0 | 0.42 | 0.49 |
| BMI | 7 | 0.25 | 0.43 |  | 0 | 0.25 | 0.43 |
| Waist circumference | 3 | 0.44 | 0.50 |  | 0 | 0.44 | 0.50 |
| Hypertension | 12 | 0.38 | 0.48 |  | 0 | 0.38 | 0.48 |
| Parental diabetes | 59 | 0.27 | 0.45 |  | 0 | 0.27 | 0.45 |
| Physical activity | 8 | 0.56 | 0.50 |  | 0 | 0.56 | 0.50 |
| Alcohol consumption | 9 | 0.32 | 0.46 |  | 0 | 0.32 | 0.46 |
| Smoking status | 4 | 0.74 | 0.73 |  | 0 | 0.74 | 0.73 |

Variables imputed using Markov Chain Monte Carlo procedures (5 imputations)
Full sample size n=5312; no missing values in variables study, age and sex

**Figure S1**: Proportional Venn-Diagram showing the overlap of the prediabetes criteria in women and men


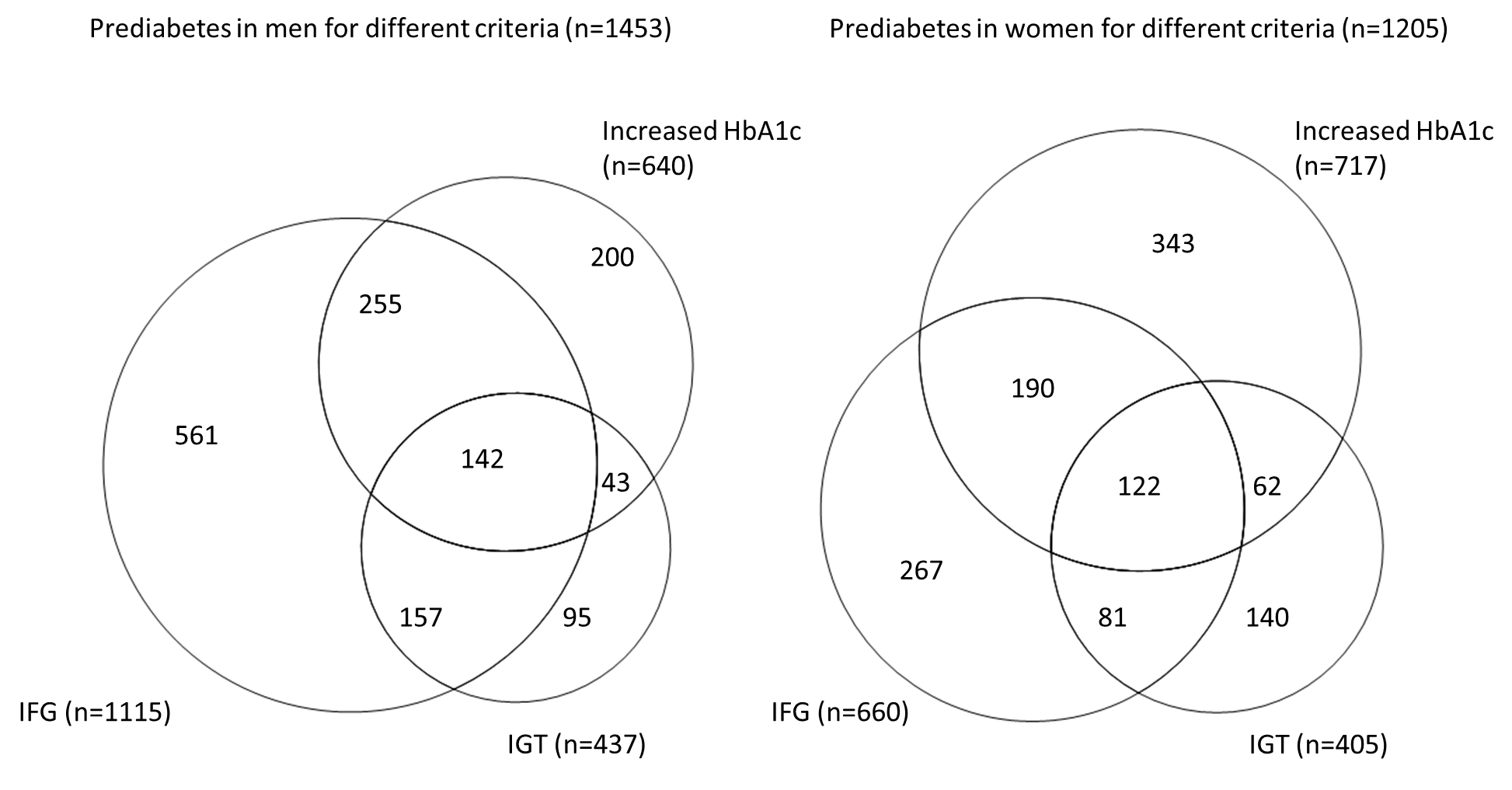

Supplement: Supplementary file 1 — Supplementary file1 (DOCX 253 kb) [file 592_2020_1573_MOESM1_ESM.docx]
